# Dihydromyricetin attenuates *Escherichia coli* lipopolysaccharide-induced ileum injury in chickens by inhibiting NLRP3 inflammasome and TLR4/NF-κB signalling pathway

**DOI:** 10.1186/s13567-020-00796-8

**Published:** 2020-05-24

**Authors:** Yicong Chang, Liang Yuan, Jiarui Liu, Ishfaq Muhammad, Chuanbao Cao, Chenxi Shi, Yuanyuan Zhang, Rui Li, Changwen Li, Fangping Liu

**Affiliations:** 1 Department of Basic Veterinary Science, College of Veterinary Medicine, Northeast Agricultural University, Harbin, China; 2 Heilongjiang Key Laboratory for Animal Disease Control and Pharmaceutical Development, Harbin, China; 3 Harbin Veterinary Research Institute of Chinese Academy of Agricultural Sciences, Harbin, China

## Abstract

Lipopolysaccharide (LPS) as a major component of *Escherichia coli* cell wall can cause inflammation and cell death. Dihydromyricetin (ampelopsin, DHM) is a natural flavonoid compound with anti-inflammatory, anti-oxidant and anti-bacterial effects. The preventive effects of DHM against ileum injury remain unclear. Here, we explored the protective role of DHM against LPS-induced ileum injury in chickens. In this study, DHM significantly attenuated LPS-induced alteration in diamine oxidase, malondialdehyde, reduced glutathione, glutathione peroxidase and superoxide dismutase levels in chicken plasma and ileum. Histology evaluation showed that the structure of blood vessels in ileum was seriously fragmented and presence of necrotic tissue in the lumen in the LPS group. Scanning electron microscopic observation revealed that the surface of the villi was rough and uneven, the structure was chaotic, and the normal finger shape was lost in the LPS group. In contrast, 0.05% and 0.1% DHM treatment partially alleviated the abnormal morphology. Additionally, DHM maintained the barrier function by restoring the protein expression of occludin, claudin-1 and zonula occludens protein-1. DHM inhibited apoptosis through the reduction of the expression of bax and caspase-3 and restored the expression of bcl-2. Importantly, DHM could reduce ileum NLR family pyrin domain-containing 3 (NLRP3), caspase-1, interleukin (IL)-1β and IL-18 expression to protect tissues from pyroptosis and inhibited toll-like receptor 4 (TLR4)/nuclear factor kappa-B (NF-κB) signalling pathway. In summary, DHM attenuated the ileum mucosal damage, oxidative stress and apoptosis, maintained barrier function, inhibited NLRP3 inflammasome and TLR4/NF-κB signalling pathway activation triggered by *Escherichia coli* LPS.

## Introduction

Avian pathogenic *Escherichia coli* (*E. coli*) is the pathogen that is associated with avian *E. coli* disease and can be transmitted through the digestive and respiratory tracts [1]. Chickens of all ages can be infected by *E. coli*, especially one-month old chicks [2]. Lipopolysaccharide (LPS), the main component of *E. coli* cell wall, is released in large quantities via bacteria or drugs mediated bacterial lysis and triggers inflammatory response and cell death [3, 4]. LPS is not only involved in the pathogenic process of *E. coli*, but also one of the main pathogenic factors of *E. coli*.

There are numerous complex microorganisms or flora in the intestine, which play a key role in development and regulation of the immune system, and prevention and control of disease in the host [5]. Moreover, because intestinal barrier function can resist pathogenic microorganisms, when the intestine is damaged, it will cause an increase in the translocation of pathogens or their toxins, thereby resulting in damage to the body. It is well known that the small intestine is the main region for nutrient absorption and it becomes one of the target organs for LPS [3]. Importantly, in addition to being involved in the pathogenesis of pathogenic *E. coli*, LPS has been shown to cause intestinal barrier dysfunction leading to increased intestinal permeability [6]. Under these circumstances, pathogenic *E. coli* or other pathogens and toxins are more likely to enter the bloodstream, causing damage to the body and eventually forming a vicious cycle. Therefore, it is vital for the body to maintain the health of the gut.

NLR family pyrin domain-containing 3 (NLRP3) is a member of the NOD-like receptor (NLR) family. Study has shown that after LPS stimulation, NLRP3 transcription increases and enters the cytoplasm to assemble with apoptosis-associated speck-like protein containing a CARD (ASC) and pro-caspase-1 to form a multi-protein complex termed the NLRP3 inflammasome that is involved in the cell innate immune defence [7]. Currently, NLRP3 inflammasome activation is reported to depend upon one, or a combination of signals that include K^+^ efflux, reactive oxygen species (ROS) generation, or destabilisation of lysosomal membranes [8]. In turn, activated caspase-1 proteolytically cleaves the cytokine precursors of interleukin (IL)-1β and IL-18 into active mature peptides and promote pyroptosis [7, 9]. In addition, NLRP3 inflammasome has been widely studied in various LPS-induced models [10].

Along with LPS-binding protein, cluster of differentiation 14 acts to transfer LPS to the toll-like receptor 4 (TLR4) [11]. After LPS activates TLR4, nuclear factor kappa-B (NF-κB) is activated through myeloid differentiation factor 88 (MyD88)-dependent and MyD88-independent signal transduction pathways [11]. Activated NF-κB enters the nucleus and modulates the induction of multiple proinflammatory cytokines, including IL-1β, IL-6, IL-8 and tumour necrosis factor-α (TNF-α) [12]. Moreover, NF-κB is involved in the occurrence of programmed cell death [13, 14].

Chinese Rattan tea [*Ampelopsis grossedentata* (Hand.-Mazz.) W.T.Wang] is a traditional tea that has many effects to promote health. Dihydromyricetin (DHM), also named ampelopsin, is a natural flavonoid compound extracted from the stems and leaves of *Ampelopsis grossedentata* (Hand.-Mazz.) W.T.Wang [15]. It exhibits multiple pharmacological effects, such as anti-inflammation, anti-oxidation and anti-bacterial effects [16, 17]. Moreover, ampelopsin possessed a strong antioxidant activity and alleviated LPS-induced oxidative stress in piglets [18]. The preventive effects of DHM against chicken ileum injury remain unclear. Therefore, this study explored the protective mechanisms of DHM against *E. coli* LPS induced ileum injury in chickens through antioxidants, alleviating intestinal lesions, inhibiting apoptosis and NLRP3 inflammasome, and analysed possible downstream targets of drug involving TLR4/NF-κB signalling pathway.

## Materials and methods

### Reagents and antibodies

*Escherichia coli* LPS (055:B5) was purchased from Sigma-Aldrich (St. Louis, MO, USA). DHM was purchased from Shanghai Winherb Medical Technology Co., Ltd. (Shanghai, China; CAS No. 27200-12-0) and was purified (purity > 98.0%) from Chinese Rattan tea by high performance liquid chromatography. Anti-claudin-1 antibody was purchased from ABclonal Technology (Wuhan, China). Anti-glyceraldehyde-3-phosphate dehydrogenase (GAPDH) antibody was purchased from Huamei Biological Engineering Co., Ltd. (Wuhan, China). Anti-bax, anti-bcl-2, anti-caspase-3, anti-occludin, anti-ZO-1, anti-TLR4, anti-NF-κB p65 and anti-phospho-p65 antibodies, HRP-labeled goat anti-rabbit IgG and HRP-labeled goat anti-mouse IgG were purchased from Bioss Biotech Co. Ltd. (Beijing, China).

### Animals and experimental protocol

One-day-old Hy-line White female chickens were obtained from Xianfeng chicken farm situated in Harbin (China). Chickens were housed in cages in a controlled environment under standard conditions with a 12-h light/dark cycle and free access to feed and water. Temperature and relative humidity were provided in accordance with the requirements of chickens.

A total of 90 chickens were randomly divided into six groups (*n* = 5) with 3 replicates: control group, LPS group, 0.025% DHM + LPS group, 0.05% DHM + LPS group, 0.1% DHM + LPS group and 0.1% DHM control group. At 8 days of age, chickens in all DHM groups were fed DHM for 14 days. At 22 days of age, chickens in all groups excluding control groups were given 60 mg/kg LPS by intraperitoneal injection (i.p.). Referring to the dose of Huang et al. [14], preliminary tests by our group found that 60 mg/kg LPS could induce ileum and liver injury in chickens. After 12 h, chickens were sacrificed by cardiac puncture and blood was collected following euthanasia with sodium pentobarbital. The plasma was collected to determine plasma diamine oxidase activity. The ileum was isolated for histopathological examination and scanning electron microscopy observation and the remaining was stored at −80 °C for detection of other indicators, real-time RT-PCR and Western blot.

### Ileum injury specific indicators detection

Diamine oxidase (DAO) activity was determined using DAO assay kit (Nanjing Jiancheng Institute of Biotechnology, China) according to the manufacturer’s instructions.

### Ileum oxidative stress indicators detection

Malondialdehyde (MDA) and reduced glutathione (GSH) concentrations and Total superoxide dismutase (SOD) and glutathione peroxidase (GSH-Px) activities in ileum were determined using MDA assay kit, GSH assay kit, Total SOD assay kit and GSH-Px assay kit (Nanjing Jiancheng Institute of Biotechnology, China) according to the manufacturer’s instructions.

### Histological analysis

The ileum tissue was fixed with 10% neutral formalin buffer for more than 24 h. After dehydration in an ascending series of ethanol and clearing in dimethylbenzene, the tissues were embedded in paraffin and sectioned transversely at 4 μm. After haematoxylin and eosin (H&E) staining, the slides were examined and photos were taken with an optical microscope (Nikon E100, Japan, 100X magnifications).

### Scanning electron microscope analysis

The ileum tissue fixed in 2.5% glutaraldehyde, washed with a phosphate buffer solution, dehydrated with a gradient concentration of ethanol, replaced with tert-butanol, and dried. After the tissue was sputter-coated with gold, it was observed and photographed using a scanning electron microscope (Hitachi S-3400 N, Japan, 200X and 2000X magnification). The above steps were completed by the electron microscopic laboratory of the Life Science Biotechnique Research Centre in Northeast Agricultural University.

### Total RNA isolation and real-time RT-PCR analysis

Total RNA was isolated from the ileum using TRIzol reagent (Takara, Dalian, China) according to the manufacturer’s protocol. The absorbance ratio of each sample at 260 nm/280 nm was between 1.8 and 2.0 with acceptable quality [19]. First strand cDNA was synthesized using PrimeScript™ RT reagent Kit with gDNA Eraser (Perfect Real Time) purchased from Takara, Dalian, China. Following the manufacturer’s protocol, after removing genomic DNA, 1 μg of total RNA in each sample was reversed transcribed into cDNA and stored at −80 °C.

Real-time RT-PCR reaction was performed with TB Green^®^ Premix Ex Taq^TM^ II (Tli RNaseH Plus) obtained from Takara, Dalian, China in a Roche LightCycler^®^ 96 instrument (Shanghai, China). The steps for thermal cycling were as follows: 95 °C, 30 s for denaturation, and then 45 cycles of PCR (95 °C, 5 s; 60 °C, 30 s). The primers are shown in Table 1. Results were expressed as relative expression of mRNA levels compared to control samples and analysed according to the 2^−ΔΔCt^ method [20]. For analysis, target genes expression of each sample was normalized to GAPDH that is a suitable household gene [21].

**Table 1:** Genes and primers used in this study

Names	Accession No.	Forward primer (5′ to 3′)	Reverse primer (5′ to 3′)	Product
GAPDH	NM_204305.1	GACGTGCAGCAGGAACACTA	ATGGCCACCACTTGGACTTT	122 bp
bcl-2	NM_205339.2	GAGTTCGGCGGCGTGATGTG	TTCAGGTACTCGGTCATCCAGGTG	92 bp
bax	XM_015290060.2	CGCAAGGTCTACGCCATCATCTC	GCAGCAGACCAGCACCAAGTAG	165 bp
caspase-3	NM_204725.1	TACCGGACTGTCATCTCGTTCAGG	ACTGCTTCGCTTGCTGTGATCTTC	166 bp
NLRP3	NM_001348947.1	GCTCCTTGCGTGCTCTAAGACC	TTGTGCTTCCAGATGCCGTCAG	150 bp
caspase-1	XM_025142104.1	GTGCTGCCGTGGAGACAACATAG	AGGAGACAGTATCAGGCGTGGAAG	179 bp
IL-1β	NM_204524.1	AGCAGCCTCAGCGAAGAGACC	GTCCACTGTGGTGTGCTCAGAATC	90 bp
IL-18	NM_204608.1	AGATGATGAGCTGGAATGCGATGC	ATCTGGACGAACCACAAGCAACTG	97 bp
IL-6	NM_204628.1	ATGGTGATAAATCCCGATGAAG	CCTCACGGTCTTCTCCATAAAC	153 bp
IL-8	HM179639.1	ACACTCCTAACCATGAACGGCAAG	CTGGCACCGCAGCTCATTCC	114 bp
TNF-α	AY765397.1	CTCAGGACAGCCTATGCCAACAAG	GCCACCACACGACAGCCAAG	178 bp
IL-10	NM_001004414.2	CAGCACCAGTCATCAGCAGAGC	GCAGGTGAAGAAGCGGTGACAG	94 bp

### Western blot analysis

As described earlier [22], ileum tissues were scraped and lysed in radio immunoprecipitation assay lysis buffer supplemented with 1 mM protease inhibitor phenylmethyl sulfonyl. The lysate was centrifuged at 12 000 r/min for 10 min at 4 °C. The supernatant was collected and the total protein concentration was determined using a bicinchoninic acid protein assay kit (Beyotime Biotechnology Co., Ltd, Shanghai). The protein concentration of each sample was adjusted to the same. Subsequently, the sample was mixed with 5 × SDS-PAGE loading buffer and boiled at 100 °C for 15 min, and then stored at −80 °C.

The proteins were separated by SDS-PAGE gel electrophoresis. The proteins were then transferred to a polyvinylidene fluoride membrane. The membrane was washed 3 times with Tris-buffered saline solution containing 0.1% Tween 20, blocked with 5% skim milk for 2 h at room temperature, and incubated with mouse monoclonal anti-GAPDH (1:2000, CSB-MA000071) and rabbit polyclonal anti-ZO-1 (1:400, bs-1329R), anti-claudin-1 (1:400, A2196), anti-occludin (1:400, bs-10011R), anti-bax (1:800, bs-0127R), anti-bcl-2 (1:800, bs-0032R), anti-caspase-3 (1:800, bs-0081R), anti-TLR4 (1:800, bs-20379R), anti-p65 (1:400, bs-0465R) and anti-p-p65 (1:400, bs-0982R) primary antibodies at 4 °C for overnight. After washing, the membrane was incubated with appropriate HRP-conjugated IgG for 1.5 h at room temperature. The protein bands were visualized with chemiluminescence reagent (ECL, Affinity Biosciences, USA) and then exposed to photographic film. The density of each band was measured using Image J software. The relative expression level of each candidate protein was calculated using GAPDH as the internal normalized control with the same calibrator.

### IL-1β and IL-18 detection

The ileum mucosa was scraped and homogenized in ice cold normal saline solution with the help of a homogenizer. Lysates were then centrifuged at 3000 r/min for 10 min and the supernatant was collected. IL-1β and IL-18 contents were measured by enzyme-linked immunosorbent assay (ELISA, Kenuodi, China) according to the manufacturer’s instructions. The samples were added into a 96-well plate and run on a microplate reader (Bio-Rad iMARKTM, Shanghai, China).

### Statistical analysis

Data were expressed as the mean ± SD. Statistical analysis was performed using one-way analysis of variance (ANOVA), followed by Duncan test, using the statistical package for social sciences (SPSS, version 19.0) software.

## Results

### Effects of DHM on LPS-induced ileum injury and oxidative stress

**Figure 1:**
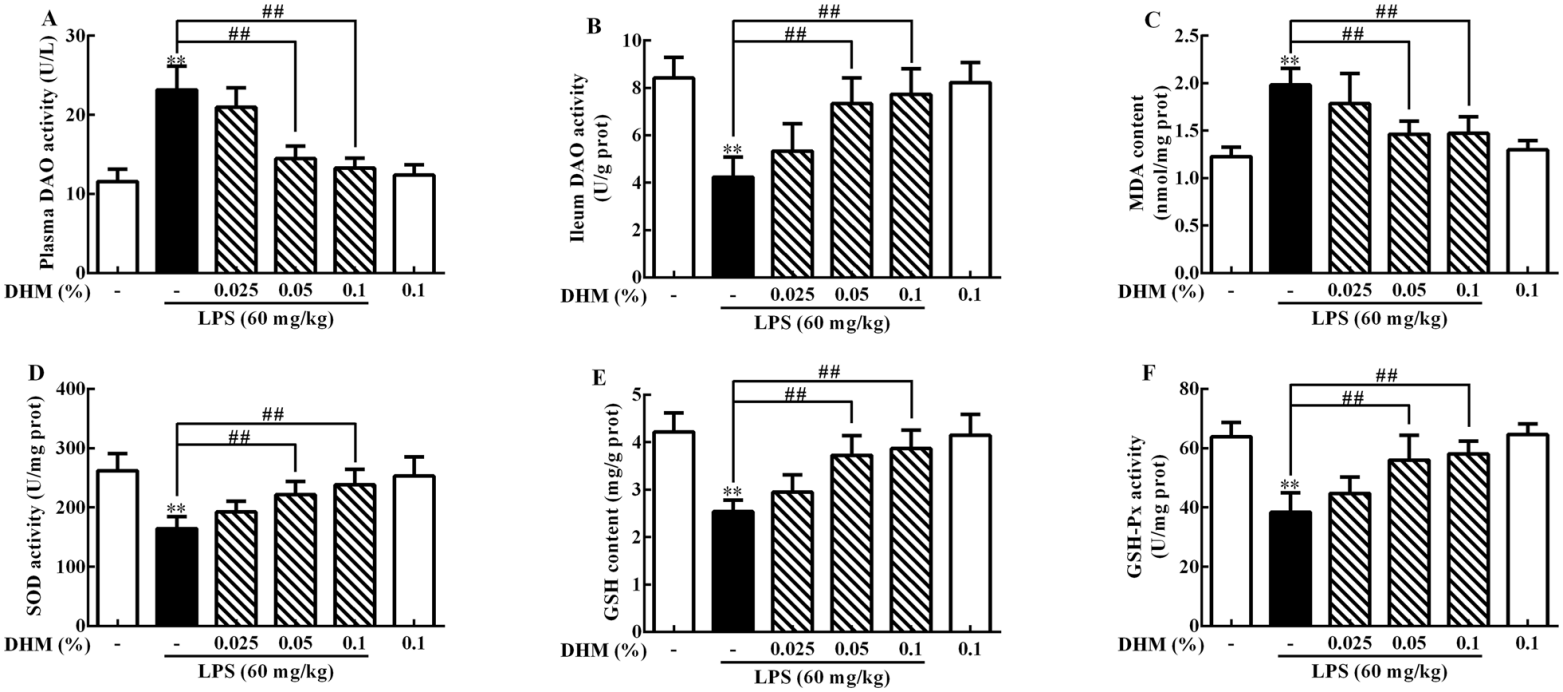
**Effects of 0.025%, 0.05% and 0.1% DHM on LPS-induced ileum injury and oxidative stress**. Changes of DAO activity (**A**) in plasma, DAO activity (**B**), MDA content (**C**), SOD activity (**D**), GSH content (**E**) and GSH-Px activity (**F**) in ileum after 60 mg/kg LPS exposure for 12 h followed by 14 days of 0.025%, 0.05% and 0.1% DHM treatment. Values are expressed as the mean ± SD for each group (*n* = 5). *p < 0.05 and **p < 0.01 represented all groups compared with the control group. ^#^*p *< 0.05 and ^##^*p *< 0.01 represented all groups compared with the LPS group.

The protective effects of DHM against LPS-induced ileum injury and oxidative stress are shown in Figure 1. Compared to the control group, LPS enhanced (*p *< 0.01) plasma DAO activity while 0.05% and 0.1% DHM reduced (*p *< 0.01) plasma DAO activity compared to the LPS group.

Compared to the control group, LPS decreased ileum DAO activity (*p *< 0.01), enhanced ileum MDA content (*p *< 0.01), reduced GSH content, SOD and GSH-Px activities (*p *< 0.01). In contrast, 0.05% and 0.1% DHM increased ileum DAO activity (*p *< 0.01), reduced ileum MDA production (*p *< 0.01), restored GSH storage (*p *< 0.01), and increased SOD and GSH-Px activities (*p *< 0.01).

### Effects of DHM on ileum pathology of chickens exposed to LPS

**Figure 2:**
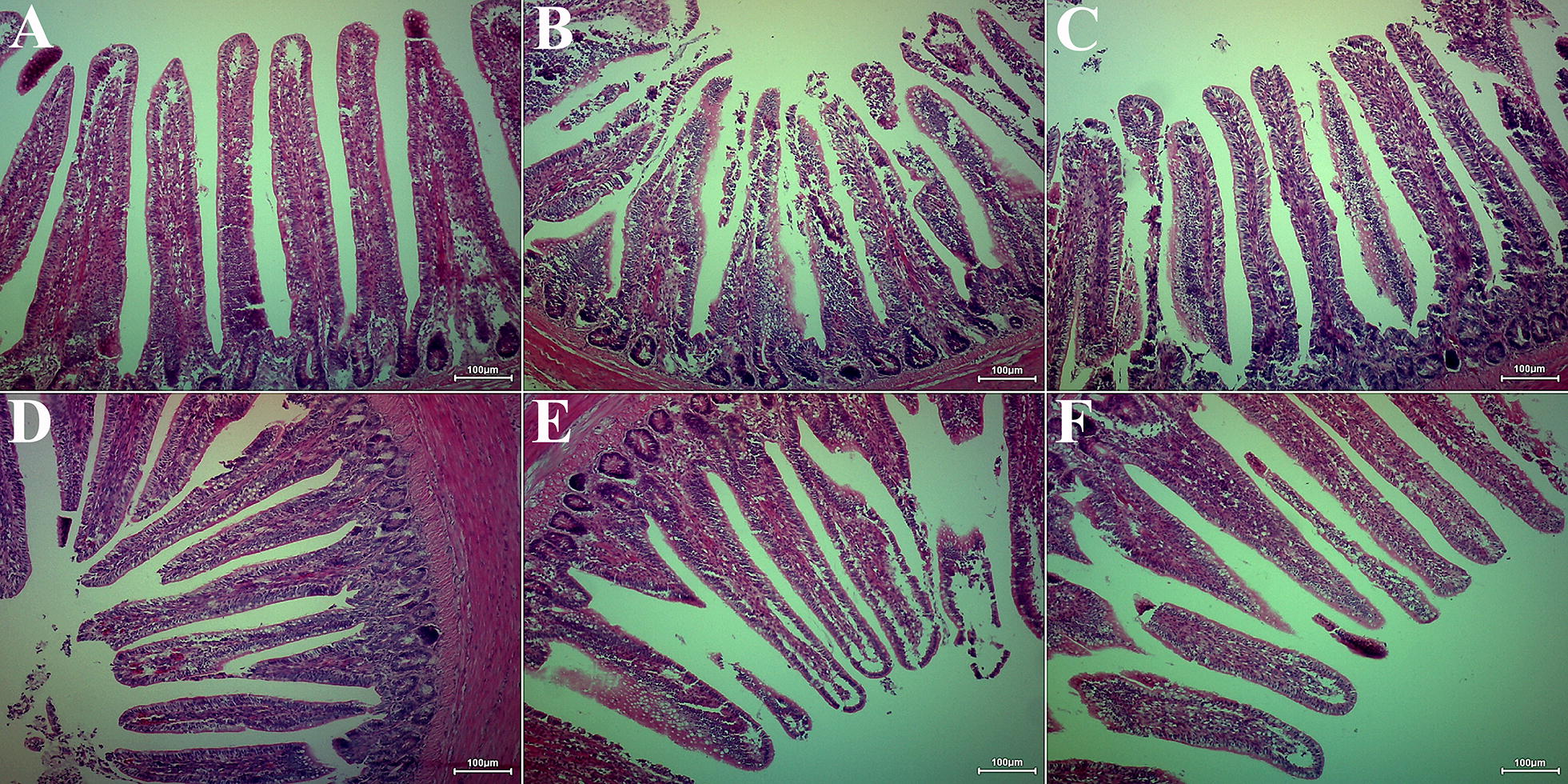
**Chickens ileum pathology**. Ileum sections were stained by haematoxylin and eosin. All the ileum sections were examined by light microscopy and the images were displayed at 100X the original magnification. (**A**) Control group; (**B**) LPS group; (**C**) 0.025% DHM + LPS group; (**D**) 0.05% DHM + LPS group; (**E**) 0.1% DHM + LPS group; (**F**) 0.1% DHM group.

The histopathological micrographs of ileum tissues measured by H&E staining are shown in Figure 2. The ileum villi structure in the control group was complete and clear in shape. In the LPS group, the intestinal villi were broken, and numerous inflammatory cells infiltrated, the structure of blood vessels was fragmented and necrotic tissue was observed in the lumen (as shown in Figure 2B). The morphology and structure of ileum villi gradually improved with the gradual increase of the preventive dose of DHM. Among them, 0.05% and 0.1% DHM (Figures 2D, E) had better preventive effects. Mucosal shedding was significantly reduced compared to the LPS group, and the morphology and structure of the mucosa were relatively normal, but there was still a small amount of inflammatory cells infiltration.

### Scanning electron microscope observation

**Figure 3:**
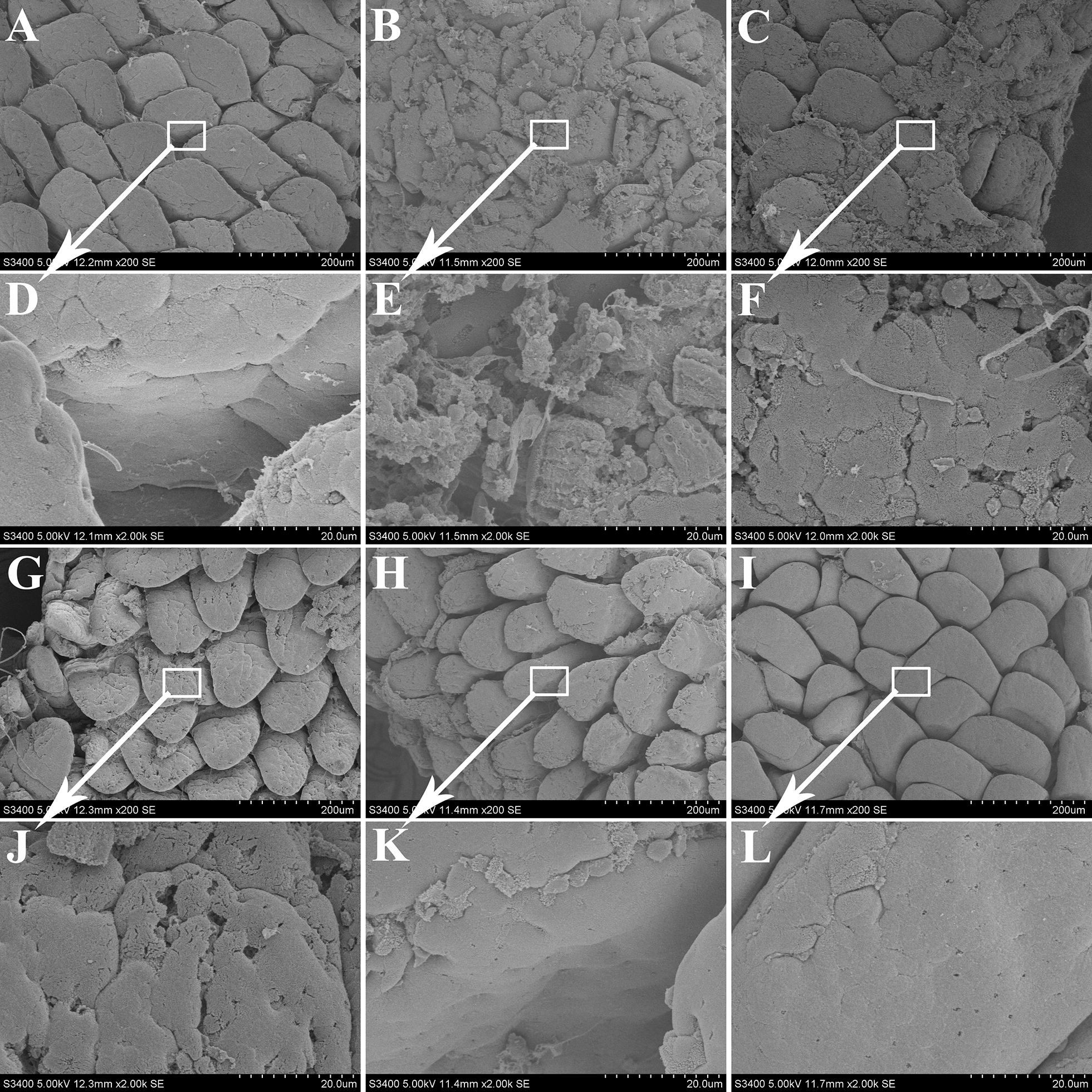
**Scanning electron microscope (SEM) observation**. SEM observation for ileum after 60 mg/kg LPS exposure for 12 h followed by 14 days of 0.025%, 0.05% and 0.1% DHM treatment. (**A/D**) Control group (200X/2000X); (**B/E**) LPS group (200X/2000X); (**C/F**) 0.025% DHM + LPS group (200X/2000X); (**G/J**) 0.05% DHM + LPS group (200X/2000X); (**H/K**) 0.1% DHM + LPS group (200X/2000X); (**I/L**) 0.1% DHM group (200X/2000X).

The observation of ileum tissue by scanning electron microscopy is presented in Figure 3. As seen from panels A and D, the intestinal villi were neatly arranged, finger-like, with wrinkles of different size on the surface, the microvilli were closely arranged and goblet cells were scattered in the control group. In the LPS group, the surface of the villi was rough and uneven, the structure was chaotic, and the normal finger shape was lost (shown in Figures 3B and E). With gradual increase of preventive doses of DHM, the microstructure of intestinal villi appeared normal. Among them, as shown in Figures 3C and F, in 0.025% DHM + LPS group, some intestinal villi returned to normal. The 0.05% and 0.1% DHM had better protection for intestinal villi than 0.025% DHM. It can be seen in Figures 3G, H, J, and K that the intestinal villus structure had basically returned to normal, but a small amount of damage was still visible.

### DHM maintained the barrier function in ileum

**Figure 4:**
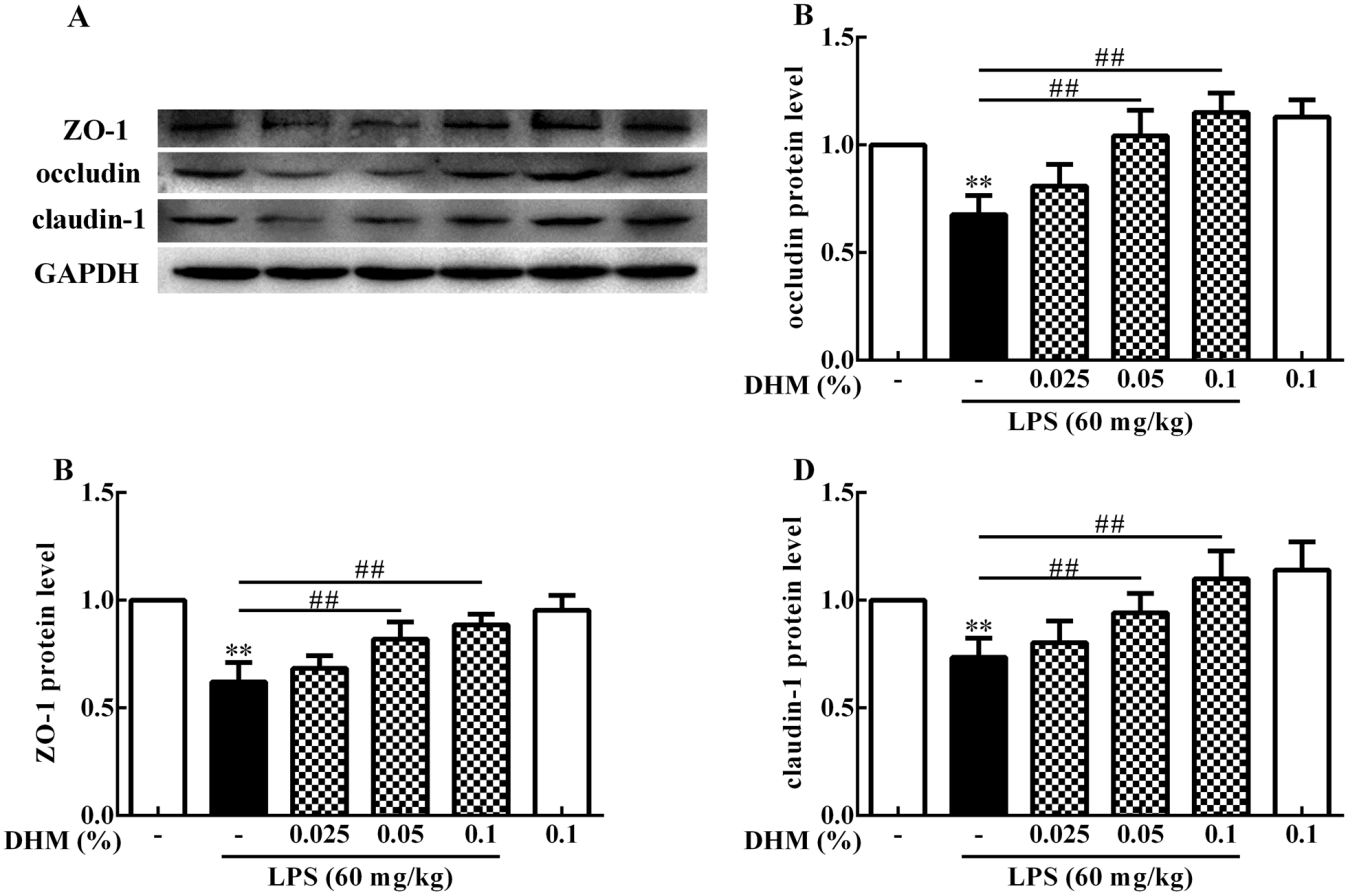
**0.025%, 0.05% and 0.1% DHM maintained the barrier function in ileum.** Original blots for ZO-1, occluding, claudin-1 and GAPDH (**A**). Changes of ZO-1 (**B**), occludin (**C**) and claudin-1(**D**) protein expression levels in the ileum after 60 mg/kg LPS exposure for 12 h followed by 14 days of 0.025%, 0.05% and 0.1% DHM treatment. Values are expressed as the mean ± SD for each group (*n* = 5). *p < 0.05 and **p < 0.01 represented all groups compared with the control group. ^#^*p *< 0.05 and ^##^*p *< 0.01 represented all groups compared with the LPS group.

To clarify the protective effects of DHM intervention for the integrity of tight junctions, ileum ZO-1, occludin and claudin-1 protein expression levels were determined (Figure 4). LPS decreased (*p *< 0.01) ZO-1, occludin and claudin-1 protein expression levels compared to the control group. Compared to the LPS group, 0.05% and 0.1% DHM increased (*p *< 0.01) ZO-1, occludin and claudin-1 protein expression levels to maintain intestinal barrier function.

### DHM resisted LPS-induced ileum apoptosis

**Figure 5:**
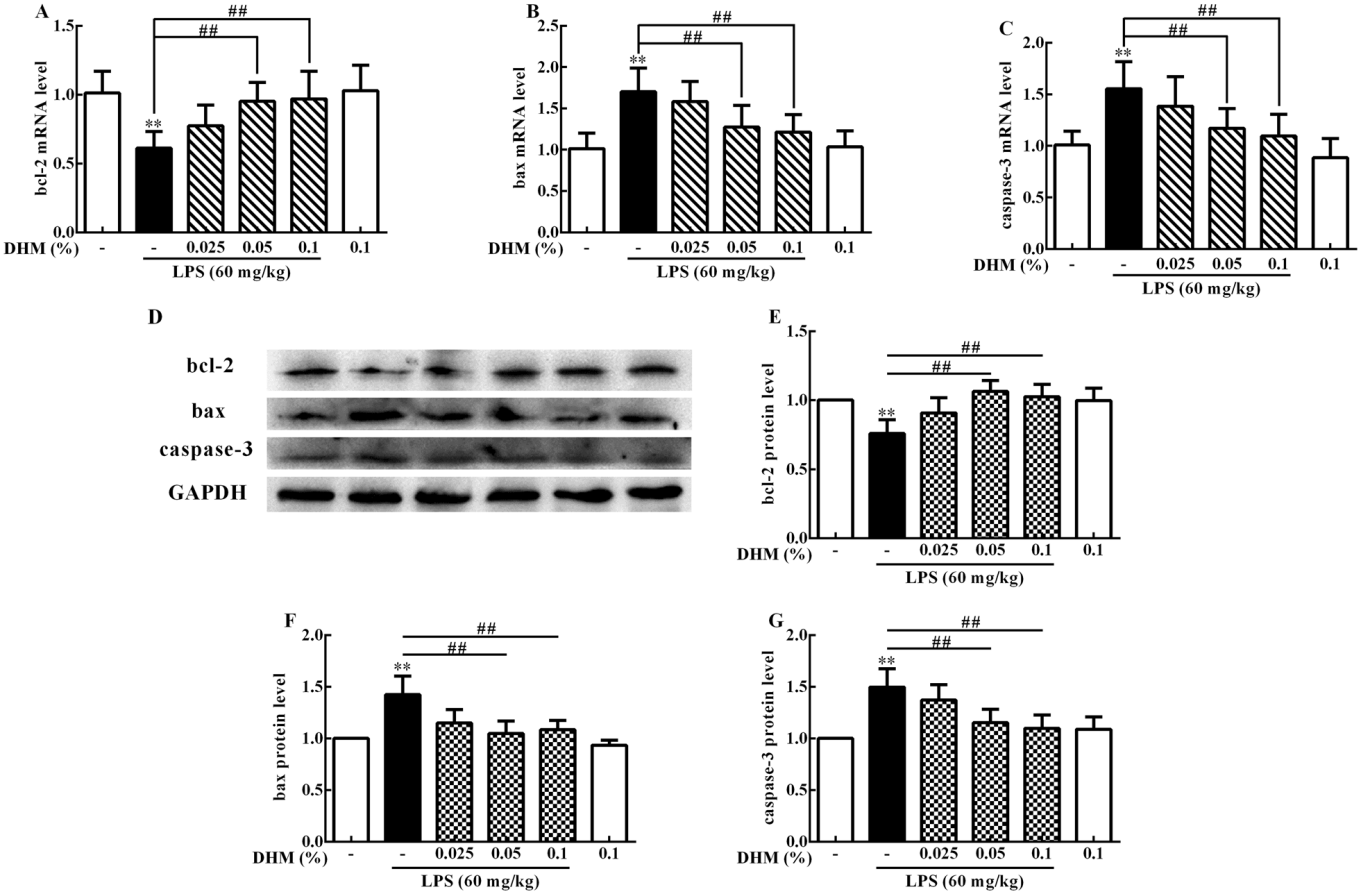
**0.025%, 0.05% and 0.1% DHM inhibited LPS-induced ileum apoptosis**. Changes of bcl-2 (**A**), bax (**B**) and caspase-3 (**C**) mRNA expression levels in ileum after 60 mg/kg LPS exposure for 12 h followed by 14 days of 0.025%, 0.05% and 0.1% DHM treatment. Original blots for bcl-2, bax, caspase-3 and GAPDH (**D**). Changes of bcl-2 (**E**), bax (**F**) and caspase-3 (**G**) protein expression levels in the ileum after 60 mg/kg LPS exposure for 12 h followed by 14 days of 0.025%, 0.05% and 0.1% DHM treatment. Values are expressed as the mean ± SD for each group (*n* = 5). *p < 0.05 and **p < 0.01 represented all groups compared with the control group. ^#^*p *< 0.05 and ^##^*p *< 0.01 represented all groups compared with the LPS group.

To investigate whether DHM protects LPS-induced ileum apoptosis, we detected bcl-2, bax and caspase-3 mRNA and protein expression levels. As shown in Figure 5, LPS reduced (*p *< 0.01) bcl-2 mRNA and protein expression levels and increased (*p *< 0.01) bax and caspase-3 mRNA and protein expression levels compared to the control group. On the contrary, 0.05% and 0.1% DHM increased (*p *< 0.01) bcl-2 mRNA and protein expression levels and inhibited (*p *< 0.01) bax and caspase-3 mRNA and protein expression levels.

### DHM inhibited NLRP3 inflammasome formation and pyroptosis activation triggered by LPS

**Figure 6:**
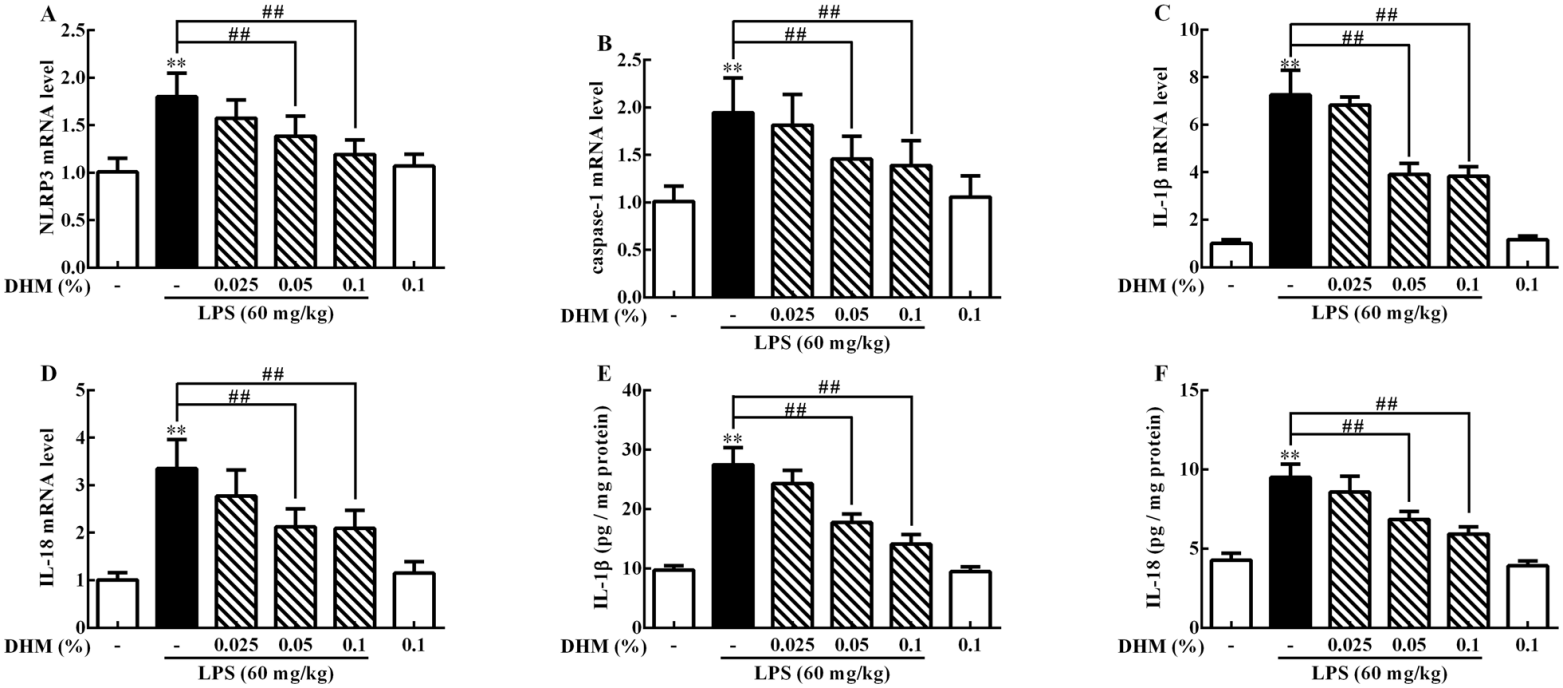
**0.025%, 0.05% and 0.1% DHM inhibited inflammasome formation and pyroptosis activation in LPS-induced ileum injury**. Changes of NLRP3 (**A**), caspase-1 (**B**) IL-1β (**C**) and IL-18 (**D**) mRNA expression levels and IL-1β (**E**) and IL-18 (**F**) contents in ileum after 60 mg/kg LPS exposure for 12 h followed by 14 days of 0.025%, 0.05% and 0.1% DHM treatment. Values are expressed as the mean ± SD for each group (*n* = 5). *p < 0.05 and **p < 0.01 represented all groups compared with the control group. ^#^*p *< 0.05 and ^##^*p *< 0.01 represented all groups compared with the LPS group.

To investigate whether DHM inhibits the formation of NLRP3 inflammasome and pyroptosis activation in LPS-induced ileum injury, we measured the mRNA expression levels of NLRP3, caspase-1, IL-1β and IL-18 and content of IL-1β and IL-18. Figure 6 showed that LPS triggered formation of NLRP3 inflammasome and pyroptosis activation relative to the control group, NLRP3, caspase-1, IL-1β and IL-18 mRNA expression and IL-1β and IL-18 contents increased (*p *< 0.01). In contrast, 0.05% and 0.1% DHM prevention inhibited the expression of NLRP3, caspase-1, IL-1β and IL-18 mRNA and IL-1β and IL-18 contents (*p *< 0.01).

### DHM resisted inflammatory response through TLR4/NF-κB signalling pathway

**Figure 7:**
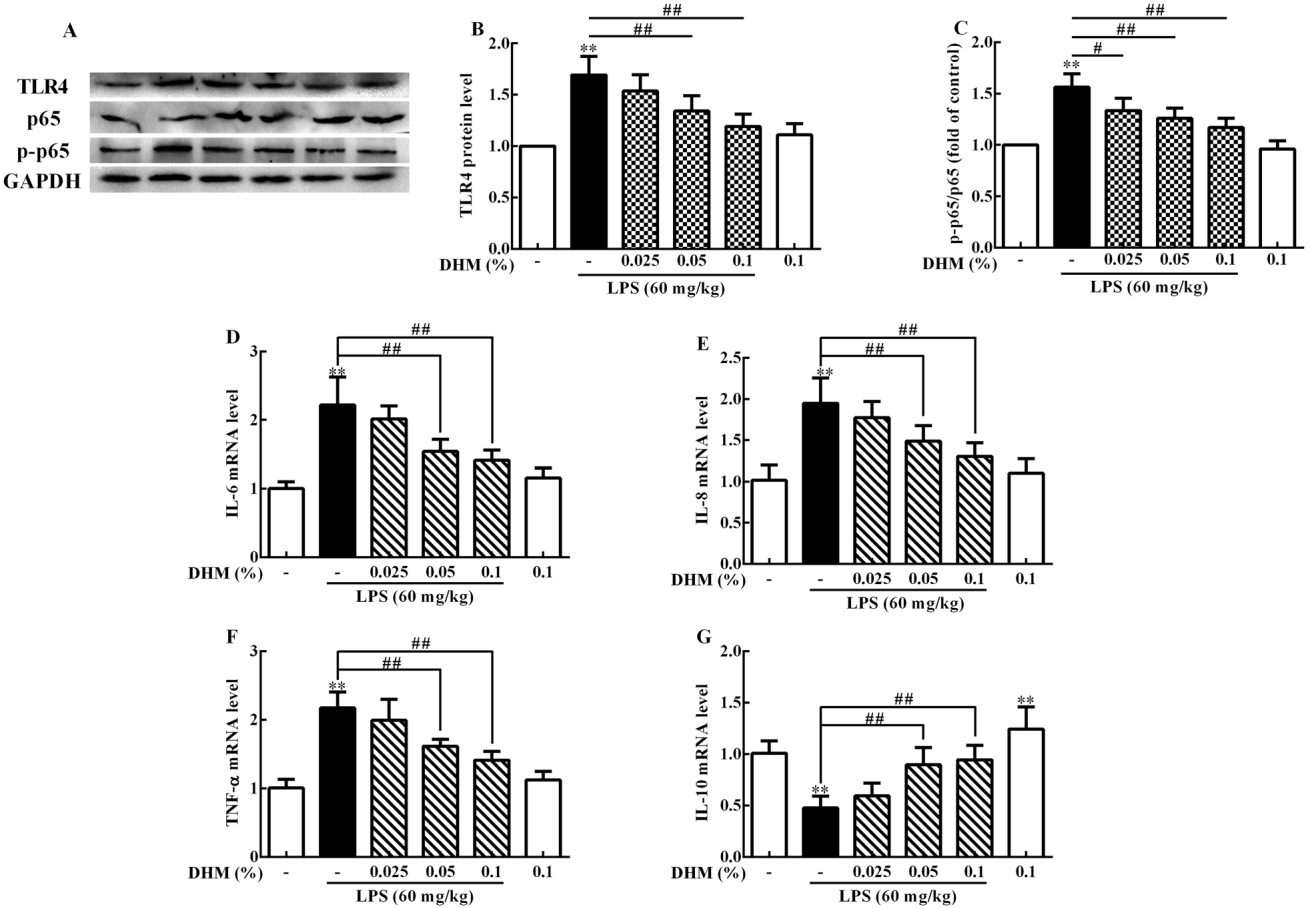
**DHM inhibited TLR4/NF-κB signalling pathway and inflammation in LPS-induced ileum injury**. Original blots for TLR4, p65, p-p65 and GAPDH (**A**). Changes of TLR4 (**B**) protein expression levels, p-p65/p65 ratio (**C**), IL-6 (D), IL-8 (E), TNF-α (**F**) and IL-10 (**G**) mRNA expression levels in ileum after 60 mg/kg LPS exposure for 12 h followed by 14 days of 0.025%, 0.05% and 0.1% DHM treatment. Values are expressed as the mean ± SD for each group (*n* = 5). *p < 0.05 and **p < 0.01 represented all groups compred with thecontrol group. ^#^*p *< 0.05 and ^##^*p *< 0.01 represented all groups compared with the LPS group.

Subsequently, we measured the protein expression levels of TLR4, the activation of NF-κB and the mRNA expression levels of IL-6, IL-8, TNF-α and IL-10. As shown in Figures 7A–C, compared to the control group, LPS caused an increase in TLR4 protein expression levels and p-p65/p65 ratio (*p *< 0.01). In contrast, 0.025% DHM reduced p-p65/p65 ratio (*p *< 0.01).

In addition, LPS induced (*p *< 0.01) the mRNA expression of IL-6, IL-8 and TNF-α, and inhibited (*p *< 0.01) the mRNA expression of IL-10 (shown in Figures 6D–G) compared to the control group. In contrast, 0.05% and 0.1% DHM could resist these inflammatory responses, reduced the mRNA expression of IL-6, IL-8 and TNF-α (*p *< 0.01), and increased the mRNA expression of IL-10 (*p *< 0.01). 0.1% DHM alone treatment markedly enhanced the mRNA expression of IL-10 (*p *< 0.01), but there was no significant effect on other inflammatory factors.

## Discussion

Although the body has some resistance to pathogenic *E. coli*, infection occurs when different stress factors cause the body’s resistance to decrease. *E. coli* disease is mainly characterized by systemic inflammation, causes severe damage to the host and even death [23]. In addition, intestinal inflammatory disease is a common intestinal tissue disease that seriously disturbs the health of livestock and poultry [24]. The main clinical symptoms such as digestive dysfunction, abdominal pain, diarrhoea and blood in the stool were noted in intestinal tissue damage [24, 25]. LPS is released in large quantities via bacteria or drugs mediated bacterial lysis and triggers inflammatory response [26]. After LPS is recognized and bound by TLR4, NF-κB signalling pathway is activated. This pathway leads to inflammatory responses and apoptosis [27].

In addition to the general properties of flavonoids, DHM has protective effects on liver ischemia–reperfusion injury, chemical liver injury and alcoholic liver diseases [28]. DHM regulates inflammation, proliferation, and down-regulation of target genes associated with TNF-α-induced apoptosis by inhibiting NF-κB activation [29]. Therefore, this study explored the effects of DHM on *E. coli* LPS induced ileum injury, oxidative stress, barrier dysfunction, apoptosis and NLRP3 inflammasome activation. It provided a vision for maintaining the intestinal health of livestock and poultry and resisting the toxic effects of LPS.

The activity of DAO in intestinal mucosa and blood putatively reflects the maturity and integrity of intestinal epithelial cells [30]. MDA, SOD, GSH and GSH-Px were the common indicators to reflect oxidative stress [31, 32]. This study showed that DHM reduced plasma DAO and caused increased ileum DAO activity. Moreover, DHM could resist oxidative stress caused by LPS, reduce MDA content and increase SOD and GSH-Px activity and GSH reserve in ileum. Similarly, both histopathological examination and scanning electron microscopic observations showed that different doses of DHM provided different degrees of protection against LPS-induced damage, of which 0.05% and 0.1% DHM had better protective effects.

Tight junctions (TJs) plays a key role in preventing the translocation of pathogenic antigens or other harmful substances from intestinal tract into circulating system [33]. Occludin, ZO-1 and claudin-1 were members of transmembrane proteins, peripheral membrane proteins and backbone proteins of TJs, respectively, which together maintained TJs integrity and intestinal barrier function [34, 35]. Some studies have shown that the significant down-regulation of TJs protein expression is the main reason for intestinal barrier dysfunction [6, 36]. LPS significantly decreased expression of TJs molecules ZO-1, occluding and claudin-1 and induced barrier dysfunction in this experiment. In contrast, DHM maintained the barrier function by increasing the expression of these proteins. Interestingly, the protein expression levels of occludin and claudin-1 were slightly increased by the action of 0.1% DHM alone (shown in Figures 4B and D), suggesting that DHM may maintain the integrity of intestinal barrier function by upregulating their expression. Similarly, histology evaluations and scanning electron microscope observations confirmed that after 0.1% DHM alone treatment, the ileum villi were arranged more neatly and the surface was appeared flatter.

Caspase-3 is a crucial apoptotic protease in the final common pathway of the apoptotic cell death [37]. Bcl-2 and bax are important regulators of apoptosis in which bcl-2 can suppress apoptosis and bax can promote apoptosis [38]. This study observed a significant decrease in the expression of the anti-apoptotic protein bcl-2 and increase in caspase-3 and the pro-apoptotic protein bax after exposure to LPS. In contrast, DHM treatment significantly inhibited LPS-induced apoptosis by upregulating the expression of bcl-2 and reducing bax and caspase-3. Additionally, when challenged with certain pathogen-associated molecular patterns, NLRP3 inflammasome promotes the activation of caspase-1 which releases proinflammatory cytokines, such as IL-1β and IL-18, and pyroptosis [7]. This study showed that DHM significantly inhibited the expression of caspase-1, down-regulated the expression and contents of IL-1β and IL-18 and seemed to reduce the activation of pyroptosis.

In addition, DHM intervention significantly inhibites the LPS-induced expression of proinflammatory mediators, such as IL-6, IL-8 and TNF-α. This anti-inflammatory effect of DHM is further supported by our findings that DHM attenuates LPS-induced TLR4 expression and NF-κB activation. In the present study, DHM alone treatment significantly up-regulated the expression of IL-10, suggesting that the anti-inflammatory effect of DHM can be attributed to the promotion of anti-inflammatory cytokines production in the ileum. Moreover, no significant changes in apoptosis and NLRP3 inflammasome related factors caused by DHM alone treatment indicated that DHM indirectly related to these factors. Studies have demonstrated that NF-κB participates in caspase3-mediated apoptosis and regulates the expression of genes involved in anti-apoptosis, bcl-2, and pro-apoptosis, bax [39, 40]. At the same time, NF-κB can also transcribe and induce NLRP3 inflammasome resulting in the occurrence of cell death [41]. Based on the results of our study, DHM reduced the NLRP3 inflammasome related factors and inflammation by inhibiting TLR4/NF-κB signalling pathway and suppressed the apoptosis by up-regulating bcl-2 expression and down-regulating bax and caspase-3 expression.

In conclusion, the present study demonstrated that *E. coli* LPS induced ileum mucosal damage, oxidative stress, barrier dysfunction, and the activation of NLRP3 inflammasome and TLR4/NF-κB signalling pathway. In contrast, DHM attenuated ileum mucosal damage and oxidative stress, maintained the barrier function, and inhibited NLRP3 inflammasome and TLR4/NF-κB signalling pathway activation. Additionally, suppression of apoptosis and promotion of anti-inflammatory factors and tight junction proteins were involved in the protective effects of DHM in ileum injury induced by *E. coli* LPS.

## Data Availability

The datasets used and/or analysed during the current study are available from the corresponding author upon reasonable request.

## Abbreviations

ASC: Apoptosis-associated speck-like protein containing a CARD
DAO: Diamine oxidase
DHM: Dihydromyricetin
*E. coli*: *Escherichia coli*
ELISA: Enzyme-linked immunosorbent assay
GSH: Reduced glutathione
GSH-Px: Glutathione peroxidase
H&E: Haematoxylin and eosin
IL: Interleukin
LPS: Lipopolysaccharide
MDA: Malondialdehyde
MyD88: Myeloid Differentiation Factor 88
NF-κB: Nuclear factor kappa-B
NLR: NOD-like receptor
NLRP3: NLR family pyrin domain-containing 3
ROS: Reactive oxygen species
SOD: Superoxide dismutase
SPSS: Statistical package for social sciences
TJ: Tight junction
TLR4: Toll-like receptor 4
TNF-α: Tumour necrosis factor-α
